# 16S rRNA gene sequencing-based preliminary study on the differences in the microbiota between children with rampant caries and those with arrested caries

**DOI:** 10.3389/froh.2026.1693174

**Published:** 2026-04-17

**Authors:** Meimei Li, WenChee Wong, Huacui Xiong, Ke Chen

**Affiliations:** Stomatological Hospital, School of Stomatology, Southern Medical University, Guangzhou, Guangdong, China

**Keywords:** 16S rRNA, arrested caries, child, dental caries, microbiota, rampant caries

## Abstract

**Objective:**

In clinical practice, arrested caries (AC) poses less harm to children than rampant caries (RC), as the development of caries is arrested. However, there is limited research on the microbiology of the two types of caries. This research study the differences in microbial profiles among AC, caries-free (CF)and RC patients.

**Methods:**

Thirty-six children aged 3–5 years were selected, grouped into AC, CF, and RC groups, with 12 children in each group. A total of 72 samples, including non-stimulated saliva and dental plaque, were collected. Microbial DNA was extracted, and the V3-V4 region of the 16S rRNA gene was sequenced using the Illumina MiSeq platform. Bioinformatics analysis was performed with QIIME2, and taxonomic classification was based on the SILVA database. Alpha and beta diversity were assessed, and the Kruskal–Wallis test (with Benjamini-Hochberg correction) was used to identify taxonomic abundance differences.

**Results:**

The α-diversity in plaque was significantly lower than in saliva. While the salivary microbiome showed minimal variation across different caries states, the plaque microbiome displayed distinct structural differences. At the taxonomic level, Bacteroidota and *Prevotella* were enriched in the RCP group, while Fusobacteriota and *Leptotrichia* were more abundant in the ACP and CFP groups, with *Corynebacterium* being most abundant in the arrested caries group. Differential abundance analysis identified five putative species-level biomarkers associated with specific clinical states in dental plaque.

**Conclusion:**

This study suggests that different caries statuses are linked to distinct microbial profiles in dental plaque. The analysis revealed clear differences in microbial community structures across the three clinical groups, highlighting a potential connection between caries activity and plaque dysbiosis.

## Introduction

According to the rate of caries progression, deciduous dental caries can be characterized as acute caries or chronic caries. Rampant caries (RC) is a typical manifestation of acute caries that occurs suddenly and develops rapidly, involves a wide range of teeth, spreads to the dental pulp in the early stage, and often occurs on teeth and surfaces that are not prone to caries, such as the labial surface of the anterior teeth. Arrested caries (AC), also known as inactive caries, are a typical manifestation of chronic caries; when the crown is widely broken, the pulp is still healthy, caries stop progressing, and the surface becomes hardened, smooth, and dark brown (1). AC is less harmful to children than RC because of the slow or static development of caries. Therefore, improving dental caries control in children has long been an important topic for dental caries researchers.

Aetiological pathogenic microorganisms in dental caries have been studied for a long time. *S. mutans* was the first bacterium identified to be associated with human dental caries (2). However, subsequent research has shown that *S. mutans* cannot be isolated from the oral cavities of some individuals with caries (3, 4). The understanding of the aetiology of dental caries has gradually changed from the theory of single pathogenic bacteria to the theory of microecological imbalance. The occurrence and development of dental caries are related to the disruption of the microecological acid‒base balance and the acidic state of biofilms (5, 6). With the continual development of modern molecular biology technology, molecular detection technology has been widely used in the study of dental caries microorganisms, and it has been confirmed that the imbalance of dental caries microecology involves a variety of microorganisms.

There are many microbiological studies on early childhood caries (ECC) in young children, but there are no studies on the rate of caries progression in children. Ma (7) used a human oral microbe identification microarray (HOMIM) to compare the saliva and plaque microorganisms of CF patients and severe early childhood caries (SECC) patients. The abundances of *S. mutans*, *Porphyromonas* spp. and *Actinomyces* spp. were strongly correlated with the SECC. In addition to *S. mutans*, other acid-producing and acid-resistant bacteria were also detected in the hyperacidic dental plaque of children with ECC. These bacteria, including *non-S. mutans*, *Actinomyces* spp., *Lactobacillus* spp., Bifidobacterium and Scardovia species, can promote the pathogenesis of this disease by enhancing the acidification of the biofilm environment (8). Using an *in vivo* dental caries model (9), reported that *Streptococcus* spp., Bifidobacterium, Atopobium, *Prevotella* spp., *Veillonella* spp. and *Saccharibacteria* (TM7) were more evenly distributed when dental caries progressed from an active to a static status according to Illumina sequencing of the 16SrRNA gene. The bacterial microbiome related to lesion progression was considered significantly different from that related to lesion arrest.

At present, there are no cross-sectional studies on the microbial community of children with RC and AC. Therefore, in this study, Illumina MiSeq sequencing was used to compare the saliva and dental plaque of CF, AC and RC children to analyse the differences in the microbiota and to study the role of the microbiota in the progression and cessation of dental caries in children; this work will reveal candidate biomarkers for future longitudinal studies to screen and evaluate the risk of caries development in children and help clinicians and patients control dental caries development.

## Materials and methods

### Research population

**Figure 1:**
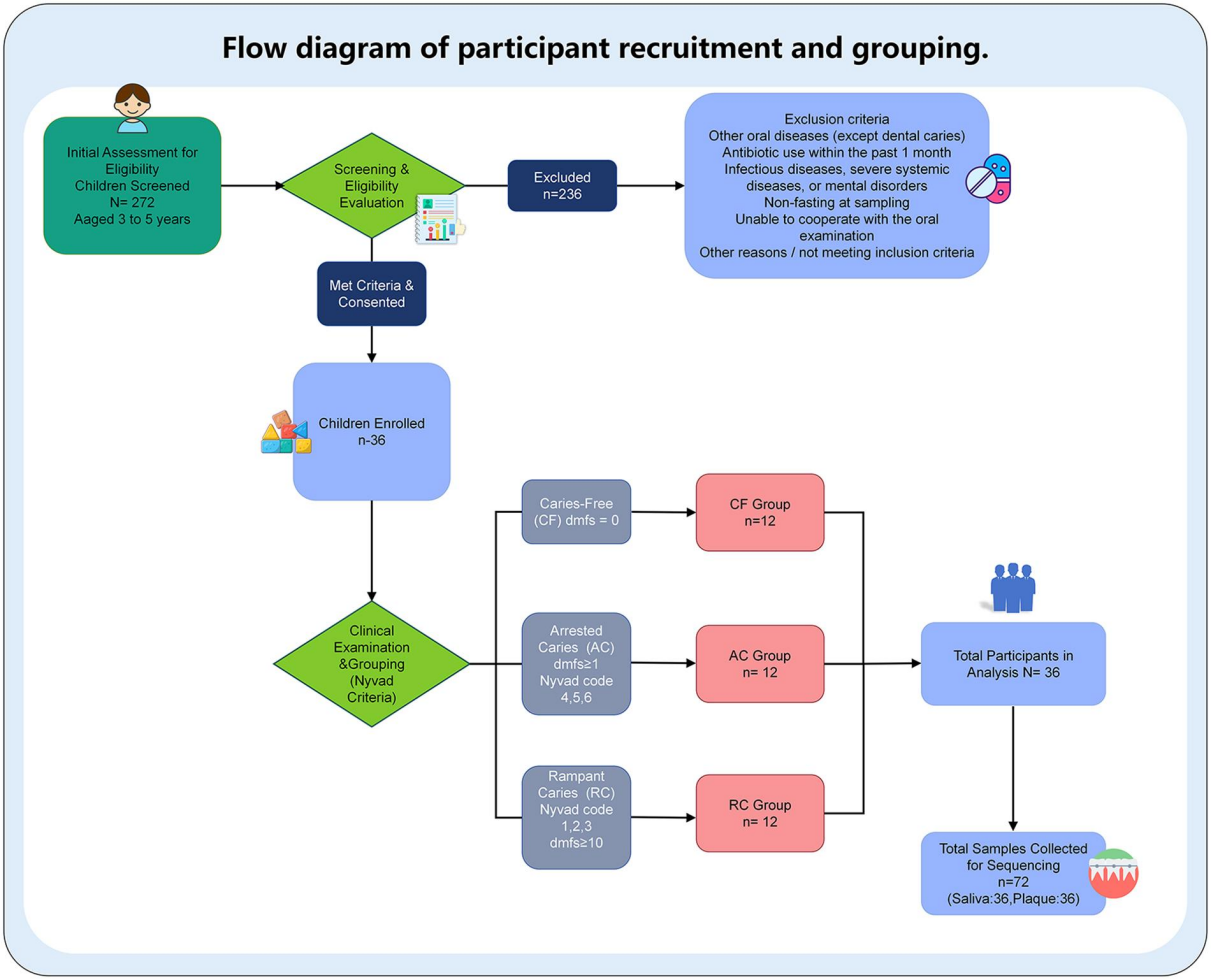
Flow diagram of participant recruitment and grouping.

This study was approved by the Ethics Committee of Stomatological Hospital, School of Stomatology, Southern Medical University [project number: Yue Kou Medical Ethics Review (2019) 02]. There were 36 children aged 3–5 years in the Department of Paediatric Stomatology, Stomatological Hospital, School of Stomatology, Southern Medical University between February and December 2019 (Table 1 presents the baseline characteristics). There were 12 children in the CF group, 12 in the AC group and 12 in the RC group, and oral informed consent was obtained from all the subjects, and written informed consent was obtained from the guardians. The recruitment, exclusion, and grouping process is shown in Figure 1.

**Table 1:** Baseline characteristics of participants.

Characteristic	CF group (*n* = 12)	AC group (*n* = 12)	RC group (*n* = 12)	Total (*n* = 36)
Sex, *n* (%)				
Male	6 (50.00)	5 (41.67)	7 (58.33)	18 (50.00)
Female	6 (50.00)	7 (58.33)	5 (41.67)	18 (50.00)
Age (years), Mean ± SD	4.25 ± 0.65	4.34 ± 0.51	4.31 ± 0.48	4.30 ± 0.54
Sample type collected, n				
Saliva:	12	12	12	36
Plaque:	12	12	12	36

CF, caries-free; AC, arrested caries; RC, rampant caries. No statistical comparisons were performed for.

baseline characteristics due to the exploratory nature of the study and small sample size.

### Inclusion and exclusion criteria

#### Inclusion criteria

1. The age ranged from 3 to 5 years.
2. Clinical examination revealed the following: CF (dmfs = 0), AC (all dental caries in the mouth had Nyvad lesion codes of 4, 5, or 6), and RC (all dental caries in the mouth had Nyvad lesion codes of 1, 2 or 3, and dmfs ≥10) (10).
3. The patients were in good health, and the colour and texture of their lip mucosa were not abnormal.

#### Exclusion criteria

1. Patients with other oral diseases (except dental caries).
2. Antibiotics were taken within 1 month.
3. Data on infectious diseases, severe systemic diseases, mental disorders or noncooperation were collected.

### Dental plaque collection

Sample collection is done in the morning. Prior to saliva and plaque sampling, all participants were instructed to refrain from food and drink for at least 2 h before their appointment. “Fasting” was defined as no milk, no juice, and no sugary foods or drinks during this period. To ensure participant comfort, small sips of plain water were permitted. In the CF group and RC group, plaque was scraped from the healthy enamel surfaces of the whole mouth with a sterile excavator. The group AC scraped plaque from the carious surfaces of carious teeth with a sterile excavator, aiming to directly investigate the characteristics of the microbial community associated with the cessation of caries progression. Then, the samples were immediately placed in a 1.5 ml sterile centrifuge tube with 1 ml of liquid thioglycolate (FT) for preservation, refrigerated at 4 °C, and then transferred to −80 °C for long-term storage within 4 h.

### Saliva collection

The three groups of children naturally spit 2–4 ml of noninduced saliva into a 15 ml aseptic centrifuge tube without any stimulation after dental plaque collection (11). The sample ID was recorded, and the samples were stored in a 4 °C refrigerator, and transferred to a −80 °C within 4 h.

### Grouping

A total of 72 samples were collected. According to the clinical diagnosis and sample type, the patient specimens were divided into 6 groups: (1) caries-free saliva (CFS), (2) arrested caries saliva (ACS), (3) rampant caries saliva (RCS), (4) caries-free plaque (CFP), (5) arrested caries plaque (ACP), and (6) rampant caries plaque (RCP) (*n* = 12).

### 16S rRNA gene sequencing

The DNA from the samples in each group was extracted according to the instructions of the QIAamp DNA Stool Mini Kit. DNA purity and concentration were assessed using an ND-1000 NanoDrop and Qubit. After DNA quantitative analysis and quality inspection, the 16S rRNA gene V3–V4 region was amplified from the DNA samples. The primers used for PCR amplification in this study targeted two highly variable regions, V3 and V4. The upstream primer sequence was F: CCTAYGGGRBGCASCAG, and the downstream primer sequence was R: GGACTACNNGGGTATCTAAT. All custom primers were synthesized and purified by polyacrylamide gel electrophoresis (PAGE), and the forward primer contained an index sequence for multiplexing. The system used for PCR amplification was as follows: three PCR amplifications were carried out for each sample using 20 μl reaction mixtures containing 10 pmol of each primer, 10 μl of KAPA HiFi HotStart ReadyMix (2×; Roche Sequencing/Roche, KAPA Biosystems), 20–60 ng DNA, with nuclease-free water to volume. The cycle reaction conditions were as follows: initial denaturation at 95 °C for 3 min followed by 25 cycles of 95 °C for 30 s, 55 °C for 30 s, and 72 °C for 30 s, and a final extension at 72 °C for 5 min in a DNA Engine thermocycler (Bio-Rad) (12). PCR products were verified on a 2% agarose gel, and the expected V3–V4 amplicon (about 460 bp) was purified using Agencourt AMPure XP beads (A63881). After library preparation (TrueLib DNA Library Rapid Prep kit), the final indexed library size was approximately 550 bp. The TrueLib DNA Library Rapid Prep kit (NGS00–1063) was used to construct sequencing libraries. PCR products with unique indexes were mixed in equal nanogram quantities and quantified using a NanoDrop ND1000 spectrophotometer (Thermo Scientific) to sequencing. Sequencing was performed on an Illumina MiSeq platform in paired-end 300 bp multiplex mode. After sequencing, image analysis, base calling, and error estimation were performed using the Illumina standard analysis pipeline.

### Statistical analysis

Raw sequencing reads were demultiplexed and quality filtered prior to downstream analysis. Feature tables and representative sequences were generated using the QIIME2 pipeline. Taxonomic classification was performed against the SILVA reference database (release 138.1). Differences in microbial community composition were evaluated using the Kruskal–Wallis test, followed by *post hoc* Tukey–Kramer–Nemenyi comparisons. Alpha diversity indices, including the Shannon index and observed amplicon sequence variants (ASVs), were compared among groups using the Kruskal–Wallis test. When a significant overall effect was detected, *post hoc* pairwise comparisons were performed with adjustment for multiple testing using the Benjamini–Hochberg method. Beta diversity was assessed based on weighted UniFrac distance matrices and visualized by principal coordinates analysis (PCoA). Statistical significance of between-group differences was tested using permutational multivariate analysis of variance (PERMANOVA), including pairwise comparisons. Differentially abundant taxa were identified using the Kruskal–Wallis non-parametric test, with *p* values adjusted for multiple comparisons using the Benjamini–Hochberg procedure. Taxa with a false discovery rate (FDR) <0.05 were considered statistically significant. To account for the compositional nature of amplicon sequencing data, differential abundance results were further validated using ALDEx2, a compositional data–aware method based on centered log-ratio transformation. In addition, LEfSe analysis was performed for exploratory validation, with an LDA score threshold of >2.0.

## Results

### Classification, identification and relative richness

**Figure 2:**
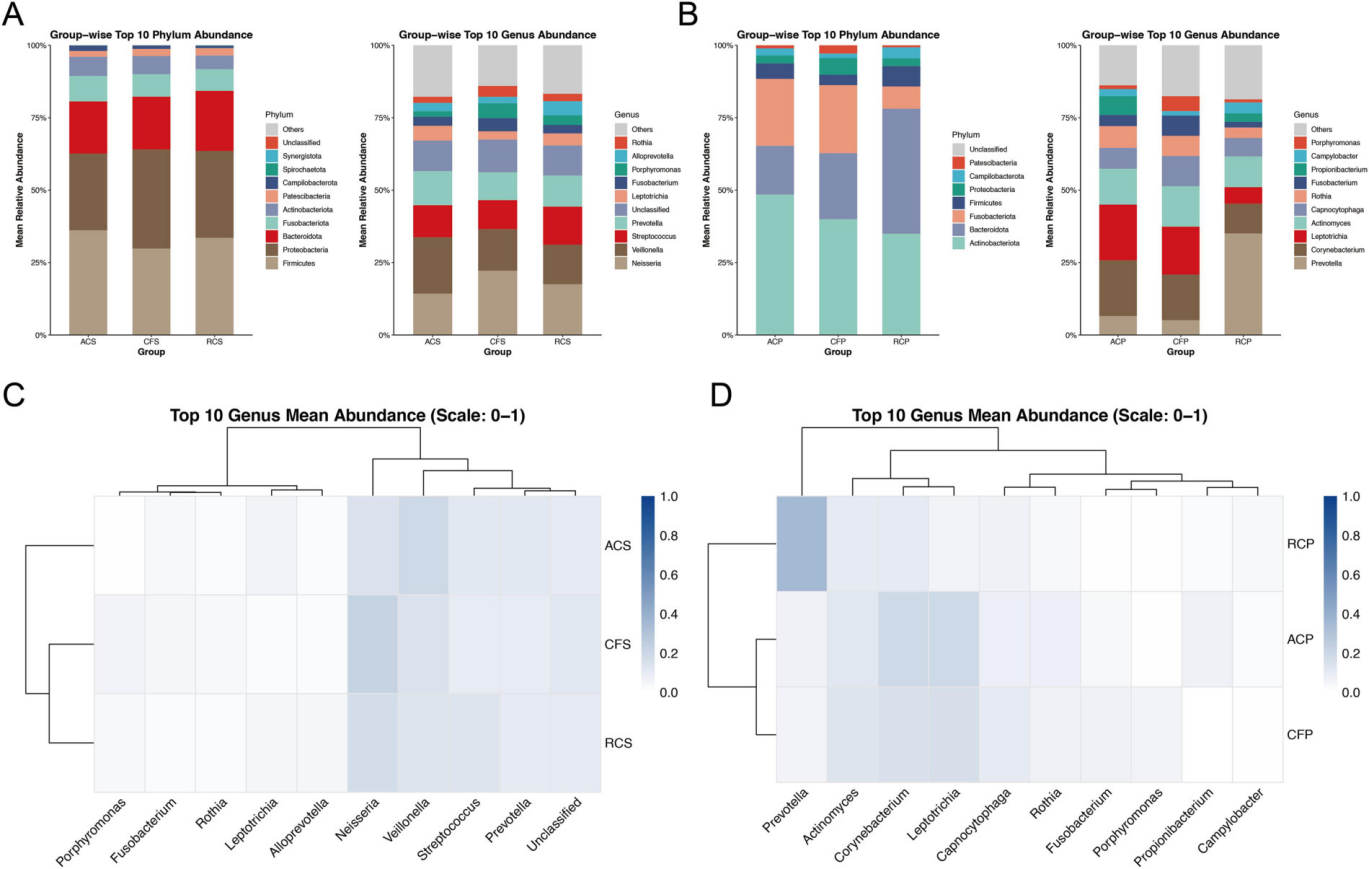
Group-wise composition of the top 10 bacterial phyla and genera in saliva and plaque samples. **(A)** Microbiodata composition at the top 10 bacterial phylum and genera in saliva samples. **(B)** Microbiodata composition at the top 10 bacterial phylum and genera in plaque samples. **(C)** Heatmap showing the mean relative abundance and hierarchicaclustering of the top 10 baterial genera in saliva samples. **(D)** Heatmap showing the mean relative abundance and hierarchical clustering of the top 10 bacterial genera in plaque samples. ACS, arrested caries saliva (*n* = 12); CFS, caries-free saliva (*n* = 12); RCS, rampant caries saliva (*n* = 12); ACP, arrested caries plaque (*n* = 12); CFP, caries-free plaque (*n* = 12); RCP, rampant caries plaque (*n* = 12).

Figure 2A presents that at the phylum level, Campilobacterota showed an overall significant difference among saliva groups, with the highest mean relative abundance observed in the ACS group and the lowest in the RCS group; however, *post hoc* pairwise comparisons did not reach statistical significance. Figure 2B presents that in plaque samples, three phyla exhibited significant differences among groups. Bacteroidota showed a higher relative abundance in the RCP group than in the ACP group. Fusobacteriota was relatively enriched in the ACP and CFP groups, while exhibiting the lowest abundance in the RCP group. In addition, Patescibacteria displayed the highest relative abundance in the CFP group and lower abundances in the ACP and RCP groups.

At the genus level, Figure 2A presents that in saliva samples, *Alloprevotella* was significantly more abundant in the RCS group than in the CFS group. Figure 2B presents that in plaque samples, *Prevotella* showed a significantly higher relative abundance in the RCP group compared with both the ACP and CFP groups, whereas *Corynebacterium* was more abundant in the ACP group than in the RCP group. *Leptotrichia* exhibited higher relative abundances in the ACP and CFP groups compared with the RCP group. In addition, *Propionibacterium* was nearly undetectable in the CFP group and showed no significant difference between the ACP and RCP groups. The genus-level abundance patterns (Figures 2C,D) shown in the heatmaps further corroborate these specific differences in both salivary and plaque microbiota.

### Alpha diversity of the microbiota

**Figure 3:**
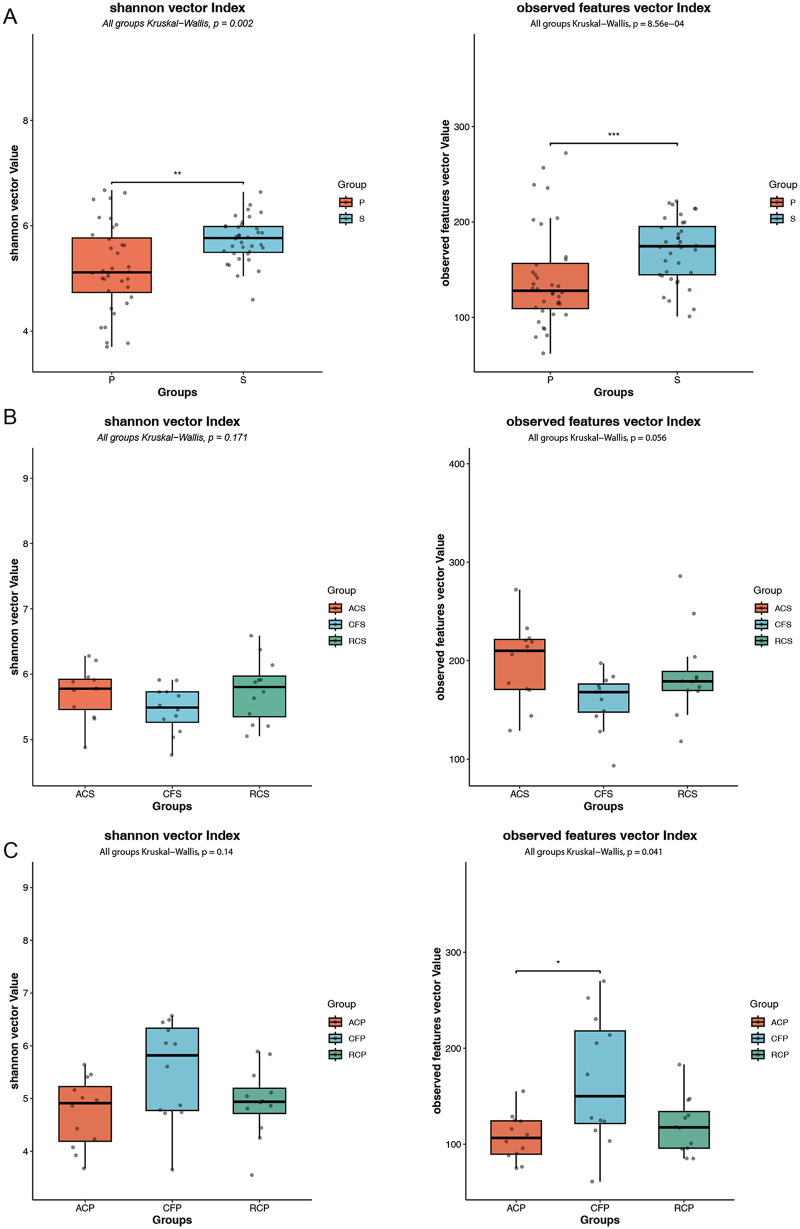
Integrated assessment of alpha diversity across saliva and dental plaque samples. **(A)** Boxplot of Shannon diversity index and Observed Features between saliva and dental plaque samples. **(B)** Boxplot of Shannon diversity index and Observed Features among saliva samples. **(C)** Boxplot of Shannon diversity index and Observered Features among dental plaque samples. P, plaque (*n* = 36); S, saliva (*n* = 36); ACS, arrested caries saliva (*n* = 12); CFS, caries-free saliva (*n* = 12); RCS, rampant caries saliva (*n* = 12); ACP, arrested caries plaque (*n* = 12); CFP, caries-free plaque (*n* = 12); RCP, rampant caries plaque (*n* = 12).

In the *α*-diversity analysis, a statistically significant difference was observed between plaque and saliva samples were shown in Figure 3A. Analysis based on the Shannon diversity index revealed a significant difference between the two sample types (Kruskal–Wallis test, *p* = 0.002). Consistently, analysis based on Observed Features further indicated that species richness in plaque samples was significantly lower than that in saliva samples (Kruskal–Wallis test, *p* = 8.56 × 10^4^).

Figure 3B presents that in saliva samples, neither α-diversity metric showed significant differences among disease status groups. Comparisons based on the Shannon index (Kruskal–Wallis test, *p* = 0.171) and Observed Features (Kruskal–Wallis test, *p* = 0.056) both yielded *p*-values above the significance threshold of 0.05.

Figure 3C presents that within plaque samples, the patterns of α-diversity across different disease status groups varied depending on the metric used. Analysis based on the Shannon index, which reflects both community richness and evenness, did not detect a significant difference among groups (Kruskal–Wallis test, *p* = 0.14). In contrast, analysis based on Observed Features, reflecting species richness, revealed a significant difference among groups (Kruskal–Wallis test, *p* = 0.041). *post hoc* pairwise comparisons using Dunn's test with Benjamini–Hochberg correction further showed that species richness in the ACP group was significantly lower than that in the CFP group (*q* < 0.05).

### Microbiota beta diversity

**Figure 4:**
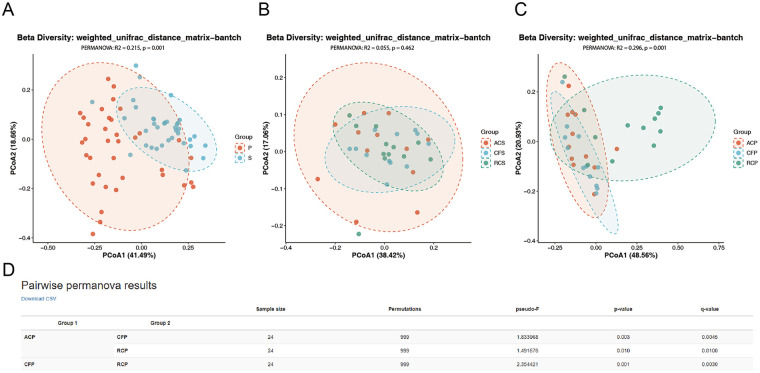
PCoA (principal coordinates analysis) plots based on weighted unifrac distance, visualizing the separation of microbial communities across sample groups. **(A)** Overall separation between saliva and dental plaque samples. **(B)** Separation among different saliva groups. **(C)** Separation among different plaque groups. **(D)** Pairwise PERMANOVA results of microbial community structure in dental plaque samples. P, plaque (*n* = 36); S, saliva (*n* = 36); ACS, arrested caries saliva (*n* = 12); CFS, caries-free saliva (*n* = 12); RCS, rampant caries saliva (*n* = 12); ACP, arrested caries plaque (*n* = 12), CFP, caries-free plaque (*n* = 12); RCP, rampant caries plaque (*n* = 12).

In the β-diversity analysis based on distance matrices, a significant difference in community structure was observed between plaque and saliva samples (PERMANOVA, *R*^2^ = 0.215, *p* = 0.001) (Figure 4A). In contrast, no significant differences in community structure within saliva samples (*R*^2^ = 0.055, *p* = 0.462) (Figure 4B). Within plaque samples, significant between-group differences were detected (*R*^2^ = 0.296, *p* = 0.001) (Figure 4C). Pairwise comparisons among all plaque groups further revealed statistically significant differences in community structure not only between the ACP and RCP groups (*q* = 0.01), but also between ACP and CFP (*q* = 0.0045) and between CFP and RCP (*q* = 0.003), with all *p*-values adjusted for multiple testing using the false discovery rate (FDR) (Figure 4D).

### Putative Species-level annotations and differential abundance analysis

**Figure 5:**
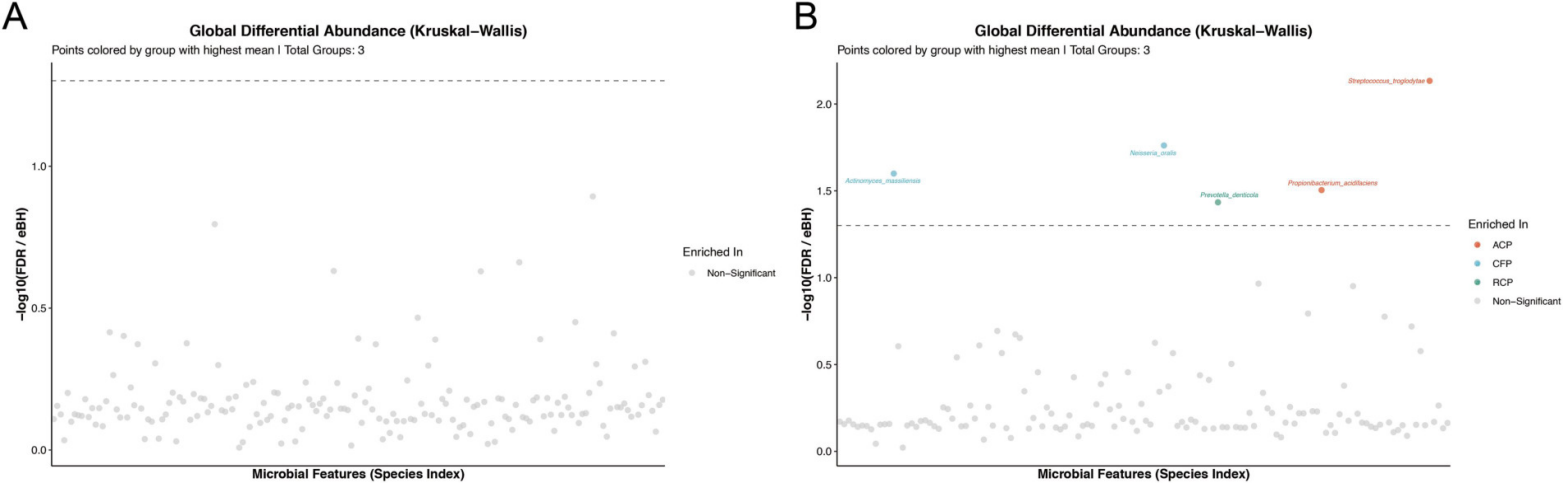
Differential abundance analysis of microbial features at the species level. **(A)** Features differing significantly among saliva groups. **(B)** Features differing significantly among dental plaque groups. Points are colored according to the group with the highest mean relative abundance see (legend). Differentially abundant taxa were identified using the Kruskal–Wallis test, with *p* values adjusted using the Benjamini–Hochberg procedure; only those with a false discovery rate (FDR) < 0.05 are labeled. Saliva groups: ACS, arrested caries saliva (*n* = 12); CFS, caries-free saliva (*n* = 12); RCS, rampant caries saliva (*n* = 12). Plaque groups: ACP, arrested caries plaque (*n* = 12); CFP, caries-free plaque (*n* = 12); RCP, rampant caries plaque (*n* = 12).

Based on 16S rRNA gene V3–V4 region sequencing data, putative species-level annotations were obtained. Differential abundance analysis showed that in saliva samples, no bacterial taxa showed significant differences among groups (Figure 5A). Most bacterial taxa did not differ significantly among plaque groups. Only five amplicon sequence variants (ASVs) exhibited statistically significant differences in relative abundance (*q* < 0.05). According to sequence similarity, these ASVs were putatively annotated as follows: *Actinomyces massiliensis* and *Neisseria oralis*, which were enriched in the CFP group; *Prevotella denticola*, which was enriched in the RCP group; and *Propionibacterium acidifaciens* and *Streptococcus troglodytae*, which were enriched in the ACP group (Figure 5B). It should be noted that these taxonomic assignments are putative, as 16S rRNA gene sequencing has inherent limitations in resolving taxa at the species level. Exploratory analysis using LEfSe (Supplementary Figure S1) revealed similar overall patterns.

## Discussion

Dental caries are related to oral microorganisms, but the types of aetiological pathogenic bacteria are not completely clear (13). The study of polymorphisms by culture methods is limited, as more than 700 kinds of bacteria have been detected in the human oral cavity, of which more than 50% cannot be cultured (14). High-throughput sequencing technology can not only enable in-depth study of the relationship between oral microorganisms and oral diseases but also fundamentally change people's understanding of the molecular basis of genetics and epigenetics underlying human health and diseases (15). Illumina sequencing is the mainstream platform in the second-generation sequencing market and has the advantages of high output, high precision, low cost, diverse applications, excellent cross-platform compatibility and diverse library preparation capabilities. Because the V3–V4 region sequenced in this study and the high sequence similarity of 16S rRNA genes among many oral bacteria limit taxonomic resolution, the sequencing data are considered to provide robust genus-level, but not universal species-level, identification. Accordingly, we mainly report taxa at the genus level, and any species-level names (e.g., *Actinomyces massiliensis*) should be regarded as putative rather than definitive assignments.

As shown in Table 1, the age and gender distributions were similar across the groups. The mean age was comparable, and the gender ratio was balanced. Therefore, age and gender are unlikely to be the primary factors contributing to microbial differences. The grouping criteria and baseline characteristics in this study may have influenced the results. The CF group was strictly defined by a dmfs index of 0, ensuring that no carious lesions were present in this group. The RC and AC groups were classified according to the Nyvad activity coding system, which distinguishes between active and inactive caries. It should be noted that the grouping in this study was based on clinical phenotypes (caries activity) rather than disease cumulative burden (dmfs index). This may have led to heterogeneity within the AC group, which could include individuals with both high and low dmfs values. Future studies could incorporate the dmfs index as a covariate to distinguish the different impacts of caries activity and cumulative history on the microbiome.

This study found that the microbial community differences related to caries status were much more pronounced in dental plaque than in saliva, which aligns with the view that dental plaque is the primary site of caries initiation (16). In dental plaque, the phylum Bacteroidota and its genus *Prevotella* were significantly enriched in the rampant RCP group. Previous studies have shown that Bacteroidota is more abundant in the plaque of caries-affected groups compared to caries-free groups (17), and *Prevotella* has higher abundance in active caries plaque compared to caries-free groups (18). The phylum Fusobacteriota and its genus *Leptotrichia* maintained higher abundance in the ACP and CFP groups, while the abundance was lowest in the RCP group. This finding is consistent with several studies, which suggest that these genera, acting as “bridges” in biofilm co-aggregation networks, are often more abundant in stable or healthy biofilms (3, 19, 20). The genus *Corynebacterium* was more abundant in the ACP group. Some species of this genus have been reported to commonly appear in carious lesions and dentin with decay (21, 22). This may be due to the fact that the ACP group was sampled from the surface of arrested caries, whereas the CFP and RCP groups were sampled from the healthy enamel surfaces. Studies have also identified Coryn*ebacterium* as a highly abundant genus in supragingival dental plaque and as an indicator of oral health (23).

In saliva, the differences in the microbiome between the groups were relatively limited. Notably, *Alloprevotella* was more abundant in the RCS group than in the CFS group. This finding is consistent with recent reports, where *Alloprevotella* was found to be associated with caries, with increased abundance in the saliva of individuals with caries (24).

The analysis revealed that microbial diversity in dental plaque is significantly lower than in saliva. Due to factors such as the flowability of saliva, extensive contact surfaces, richer nutrient and oxygen supply, temporary colonization of microbial communities, and broader sampling coverage, bacterial diversity in saliva samples is typically higher than in dental plaque (7, 25). Within dental plaque, the impact of caries status on species richness is relatively limited. Although Observed Features, which represent species count, indicated that the species richness in the ACP group was significantly lower than that in the CFP group, this may be due to the fact that the ACP group was sampled from the surface of arrested caries, whereas the CFP and RCP groups were sampled from healthy dental plaque. The species richness on carious surfaces tends to be lower than on healthy tooth surfaces (26). Furthermore, the α-diversity of the saliva microbiome was not significantly affected by caries status, which is consistent with most studies and further indicate the stability of saliva as a representative sample of the overall oral microbiome (24, 27).

β-diversity analysis revealed the distribution characteristics of oral microbiome community structure under different caries statuses. The analysis showed significant differences in the microbiome community structure between dental plaque and saliva samples, which was expected (19) and primarily attributed to the distinct locations of the two sample types. Further analysis revealed that within dental plaque, there were overall differences in microbial community structure among the different caries status groups, with significant distinctions observed between the CFP group, ACP group, and RCP group. The differences between the CFP and RCP groups further indicate the idea that caries development is associated with microbial dysbiosis (28). Additionally, plaque in the ACP group was collected from arrested carious surfaces, whereas plaque in the CFP and RCP groups was collected from sound enamel surfaces. Accordingly, the significant differences observed between ACP and both CFP and RCP likely reflect, microhabitat-specific community differences between arrested lesion surfaces and healthy enamel (9). In contrast, the community structure in saliva samples did not show significant differences, further indicating the idea that saliva is relatively insensitive to specific changes in local caries status on tooth surfaces. This highlights the greater significance of dental plaque as a sample type in caries microbiology research, compared to saliva (27, 29).

Differential abundance analysis based on 16S rRNA gene V3–V4 region sequencing revealed that, in dental plaque samples, most bacterial taxa did not show significant differences between the different caries status groups. Only five amplicon sequence variants (ASVs) exhibited statistically significant changes in abundance. This suggests that, under the cross-sectional design of this study, microbial differences between caries statuses are more likely to reflect dysbiosis of caries-associated taxa rather than a systemic reshaping of the entire plaque community (30). For these five ASVs, we performed species-level inferred annotations based on reference databases. However, it is important to emphasize that V3–V4 amplicon data have inherent limitations in species resolution, and the classification results may vary depending on the database and algorithms used. Therefore, these species identifications should be considered as indicative clues rather than definitive conclusions (31).

In contrast, no significant inter-group differences were detected in saliva samples, suggesting that the microbial signals related to caries status in this study are mainly confined to dental plaque as a localized niche (27, 29). The exploratory analysis from LEfSe showed a general trend consistent with the differential abundance analysis and can serve as complementary reference, though its interpretation also needs to consider the limitations of amplicon sequencing and cross-sectional study design (1, 32).

This study is exploratory in nature, using next-generation sequencing to reveal microbial profile differences among AC, CF and RC children for the first time. These findings offer new hypotheses about the microbial ecology associated with dental caries. However, there are several limitations. The small sample size may limit statistical power and generalizability, while the cross-sectional design prevents causal inference between microbial differences and caries development. Another limitation is the variation in dental plaque sampling sites; plaque from CF/RC groups and the AC group were collected from sound enamel and arrested carious surfaces, respectively, which could contribute to the observed microbial differences. Given these limitations, the current findings primarily highlight microbial composition differences without confirming their roles in caries development. Future longitudinal studies with larger sample sizes, incorporating metagenomic, metabolomic, and *in vitro* analyses, are needed to validate the functional roles of these bacteria in caries pathogenesis.

## Conclusion

This study reveals specific associations between different caries statuses (arrested caries, caries-free, and rampant caries) and dental plaque microbial community characteristics. The primary differences were observed in dental plaque rather than saliva samples. In the rampant caries group, *Bacteroidota* and *Prevotella* were significantly enriched, while *Fusobacteriota* and *Leptotrichia* were more abundant in the caries-free and arrested caries groups, with *Corynebacterium* being most abundant in the arrested caries group. This study was cross-sectional, and species annotations were based on 16S rRNA gene V3–V4 region sequencing, which has inherent resolution limitations. The findings indicate an association between caries activity and microbial dysbiosis in dental plaque. Future longitudinal studies integrating multi-omics approaches are needed to validate the functional mechanisms of key microorganisms.

## Data Availability

The datasets presented in this study can be found in online repositories. The names of the repository/repositories and accession number(s) can be found below: https://www.ncbi.nlm.nih.gov/, http://www.ncbi.nlm.nih.gov/bioproject/1156306.
