# Supplementary figures and images for: 16S rRNA gene sequencing-based preliminary study on the differences in the microbiota between children with rampant caries and those with arrested caries
Source: Front Oral Health. 2026 Apr 17;7:1693174. doi: 10.3389/froh.2026.1693174 (PMC13133036; doi:10.3389/froh.2026.1693174)

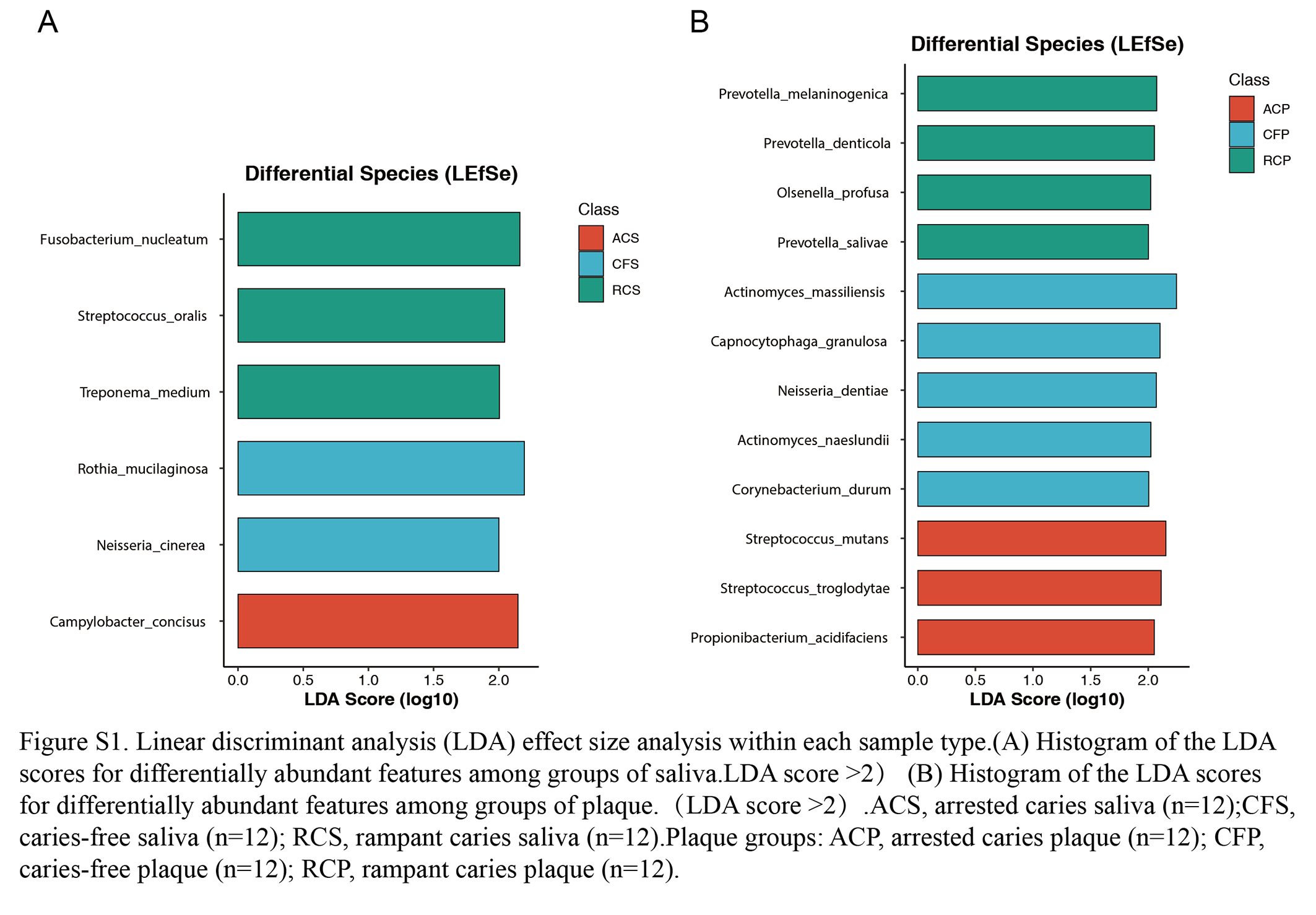

Supplement: Supplementary file 5 [file Image1.tif]
